# How Does the Duration of an Undergraduate Research Training Program Impact the Development of Professional Research Skills, Student Engagement, Sense of Belonging, and Academic Outcomes?

**DOI:** 10.3390/bs16071253

**Published:** 2026-07-22

**Authors:** Kim-Phuong L. Vu, Erin H. Arruda, Chi-Ah Chun, Panadda Marayong, Jesse Dillon

**Affiliations:** 1 Department of Psychology, California State University, Long Beach, CA 90840, USA; erin.arruda@csulb.edu (E.H.A.); chi-ah.chun@csulb.edu (C.-A.C.); 2 Department of Mechanical and Aerospace Engineering, California State University, Long Beach, CA 90840, USA; panadda.marayong@csulb.edu; 3 Department of Biological Sciences, California State University, Long Beach, CA 90840, USA; jesse.dillon@csulb.edu

**Keywords:** undergraduate research experience, duration of research training program, accelerated research training program, graduate school preparation

## Abstract

Formal undergraduate research training programs are typically two years in length to support student development over time. However, some evidence suggests that many benefits of research participation can be achieved in a shorter duration, raising the question of whether shorter models can be as effective. This prospective study compared outcomes for two concurrent cohort-based research training models: a two-year Scholars Program and an accelerated one-year Fellows Program. The Fellows Program was designed for advanced students who joined the training program later than their peers for a variety of reasons. Both programs provided students with financial support, faculty-mentored research, a Learning Community, and other resources for professional development. The longer duration allowed Scholars to report significantly higher levels of research skills and produce more publications and professional presentations than Fellows by the end of their respective training programs. In contrast, the programs did not differ on graduation GPA, number of awards, or graduate school enrollment. Fellows also did not significantly differ from Scholars on psychosocial measures, including science/researcher identity, belongingness, cultural compatibility, family support, or time management. Findings suggest that a high-intensity, one-year model can deliver many broad academic and social benefits associated with the two-year model, while a longer two-year model provides students with more time and additional research opportunities to be more productive. We conclude that both types of programs should be available to best meet students’ needs and to make the most effective use of program resources.

## 1. Introduction

Undergraduate research training is a high-impact practice associated with many positive student outcomes, particularly in Science, Technology, Engineering, and Mathematics (STEM) fields, where attrition is a major concern (DeChenne-Peters et al., 2023; Ives et al., 2024). Research shows that undergraduate research participation is linked to increased persistence in science (Jones et al., 2010; Schultz et al., 2011), higher grade point averages (Sell et al., 2018), stronger development of research skills (Gilmore et al., 2015; Vu et al., 2023), increased likelihood to graduate (Haeger et al., 2024), and shorter time to graduation (Rodenbusch et al., 2016).

Undergraduate research experiences can be obtained through several mechanisms. First, it can be offered through course-based undergraduate research experiences (CUREs), where students engage in targeted activities that incorporate discovery, use of scientific methods and research skills, collaboration, and iteration (see, e.g., Bennie et al., 2025; DeChenne-Peters et al., 2023; Ruth et al., 2023). CUREs are often considered to be a good method for providing research experiences to a greater number of students. Second, undergraduates can participate in research within faculty labs, often called faculty-mentored research experiences. This apprentice-style approach allows students to develop more focused research skills through practice and feedback while working with more experienced researchers (e.g., faculty, postdoctoral scholars, graduate students, or advanced undergraduates). However, because faculty labs have limited capacity, the number of students admitted is typically smaller than the number who can be reached through CUREs. These opportunities are not mutually exclusive, and some students engage in both types of research experiences.

Undergraduate research training can also occur through formal programs. Short, intensive research training experiences, such as the National Science Foundation (NSF) Research Experiences for Undergraduates (REU) programs, often take place over eight to twelve weeks during the summer and require full-time commitment. Participants are often early in their research development, focusing on building interest and foundational skills through hands-on experiences. However, the brief duration limits opportunities for sustained one-on-one mentorship, development of long-term post-graduation goals, and deeper mastery of research skills. These latter experiences can be obtained in other types of formal mechanisms. These include training programs historically funded by the National Institutes of Health (NIH): Maximizing Access to Research Careers (MARC), Research Initiative for Scientific Enhancement (RISE), Building Infrastructure Leading to Diversity (BUILD), and more recently, the Undergraduate Research Training Initiative for Student Enhancement (U-RISE).

A common aim of these NIH-funded initiatives has long been to broaden undergraduate participation in biomedical and behavioral research through structured training that combines faculty-mentored research with additional components designed to enhance research skills and promote professional development (see, e.g., Davidson et al., 2017; Hall et al., 2016; Hurtado et al., 2009; Plunkett et al., 2014). These programs are designed to provide supportive research training environments that help students complete their baccalaureate degree, apply for graduate school, and even transition to graduate school. The cost of these programs can be high, making opportunities competitive and limited (Cho et al., 2025; Vu et al., 2023). Moreover, these intensive research training programs often involve 2 years of participation, which can limit the number of students who are able to commit to the entire duration of the program. However, the 2-year model is often considered to be the gold standard because of its documented success in preparing students for admission into graduate programs (Hall et al., 2016; Vu et al., 2023).

Because there are many pathways to engage in undergraduate research training and the actual experience can differ substantially in structure (formal program; informal research participation), setting (course-based or faculty lab-based), and duration (days, weeks, semesters, single year, multi-year), there is major variation in what students actually experience, what supports students receive, and the length of research engagement students experience. This variability creates a challenge for research and evaluation since important summative outcomes are compared both within and between studies, but those outcomes may be dependent on the length of a research training program. Length of program participation may be an important factor due to the increased opportunity of more research activities and possibly deeper engagement across time (Chamely-Wiik et al., 2023; DeChenne-Peters et al., 2023). However, research participation is often treated as binary (e.g., participated/did not participate) in evaluation studies (e.g., Arruda et al., 2025; Sell et al., 2018), and often duration is a variable that is not explicitly accounted for or considered, perhaps since most programs offer a cohort model with a fixed, single timeframe.

Despite limited attention to duration, some research suggests that the duration of the research experience matters. DeChenne-Peters et al. (2023) found that students participating in a full-semester CURE reported it was a better way to learn about the research process than students who participated in shorter CURE modules. At the same time, other outcomes did not differ by length of participation, suggesting that a single semester may not be sufficient for broader impacts to emerge. In fact, Rodenbusch et al. (2016) found that three semesters were needed for a longer-term impact. They evaluated a freshman program and found that students who completed the full three-semester sequence of the program were significantly more likely to earn a STEM Bachelor’s degree and graduate within six years than matched peers. However, this study did not consider longer-term participation beyond three semesters or more distal outcomes such as post-graduate education.

Consistent patterns appear in other studies that compare levels of research engagement. Jones et al. (2010) found that the number of terms a student was engaged in research participation was strongly associated with retention in college. In addition, Chamely-Wiik et al. (2023) found that certain beneficial outcomes (e.g., higher graduation GPA and reduced time to degree) were associated with research experience length. Their study compared outcomes for “novice” students (1–2 semesters) and “experienced” students (≥3 semesters or >2 semesters plus a summer research experience) along with matched control groups matched on GPA, STEM major and number of credits. The experienced research students had higher graduating GPAs compared to their matched control students. Both experienced and novice research students had shorter times to degree completion than their matched controls, yet only experienced research students reported they were more likely to pursue graduate school. While longer duration appeared to be associated with greater educational achievement, findings should be interpreted cautiously, given the lack of attention to demographic factors when matching or using those variables as statistical controls, since factors like race/ethnicity and gender are also related to educational outcomes. A study of graduate students by Gilmore et al. (2015) examined the longer-term effect of the duration of undergraduate research training on the level of research skills of graduate students. Research skill performance in first-year graduate students was assessed using rubric scores assigned to a research proposal. Graduate students with undergraduate research experience scored higher on most proposal components compared to those without such prior research experience. In addition, the number of semesters of undergraduate research participation was positively correlated with higher proposal scores.

Research also indicates that perceived gains in research skills may develop over time. Adedokun et al. (2014) surveyed undergraduate students in a year-long research program about perceived gains in research skills and understanding of research processes at multiple time points throughout the year. Results showed fewer statistically significant gains at the end of the summer research component of the program than at the end of the entire program. That is, some perceived gains, such as initial confidence or understanding of research processes, emerged early in the research experience, while other gains, such as specific research skills, required sustained engagement in research to develop.

In fact, Vu et al. (2023) found a non-linear trajectory in perceived gains of research skills over time. They surveyed students for gains in research understanding and skills at four time points during a 2-year program: (1) before starting the program, (2) after the initial summer component, (3) at the end of the first year in the program, and (4) at the end of the second year in the program. Results showed different trajectories across skill domains. Some skills, such as writing, improved steadily over time, whereas data analytics and statistical skills followed a non-linear pattern: peaking after the summer component, declining by the end of the first year (possibly reflecting early overestimation of skill gain), and then increasing again by the end of the two-year program. For evaluation, these findings suggest that shorter programs may not only yield smaller gains but may also be assessed too early to capture outcomes that emerge later in the research training process.

Long-term engagement in research also shapes students’ attitudes about research and perceived research self-efficacy. In a survey of over 500 undergraduate students, Hill et al. (2022) found that students who reported two or more research experiences had higher levels of research interest and research self-efficacy than those with only one or no prior research experience. Time is also critical for the development of a scientist or researcher identity, especially for students from historically underrepresented communities in STEM. Identity development unfolds gradually and benefits from repeated opportunities to reflect on the meaning of research experiences, to consider who they are and wish to become, and to integrate the emerging scientist/researcher identity with their other core identities (Mendoza et al., 2025). A well-developed scientist/researcher identity is often attributed as a key element for STEM retention and persistence (Schwartz et al., 2010).

In sum, across both course-based and extracurricular research experiences, longer duration participation is generally associated with stronger attitudinal, learning, and both proximal and distal academic outcomes (Adedokun et al., 2014; Chamely-Wiik et al., 2023; DeChenne-Peters et al., 2023; Gilmore et al., 2015). However, these studies do not clearly disentangle whether these beneficial outcomes can be attributed to duration alone (i.e., length of time) or to training intensity (e.g., number of activities, cumulative experiences), as the two aspects are often confounded. Decoupling of duration and intensity is important for the design and evaluation of research training programs. Research designs that hold intensity constant while varying duration, such as parallel cohort models, could offer more rigorous evidence for the unique contribution of time spent in research training.

The BUILD Program at California State University Long Beach (CSULB) implemented two cohort models of undergraduate research training: a two-year Scholars Program (junior–senior year) and a one-year Fellows Program (senior-year only) during Phase 2 of their NIH-funded award (2019–2023). Both programs provided students with paid faculty-mentored research experiences paired with a structured Learning Community (LC) and professional development activities to prepare for research careers and graduate study. The main difference between the two programs was the duration of the program: Scholars followed a two-year model spanning their junior (3rd year level) and senior (4th year level) years. Fellows followed a one-year model for seniors with accelerated training for research-experienced students (see Table 1).

Because the two programs are comparable in terms of content, financial and mentoring support, and intensity of programmatic research training, but differ in duration, meaningful comparisons can be made between the longer Scholars program (two years) and shorter Fellows Program (one year). We acknowledge that duration is correlated with the total amount of training provided and therefore does not represent a purely time-based variable. Nevertheless, if time itself is necessary for development and growth, then there should be an effect of duration, indicating that interventions need to take the role of time in fostering student outcomes into account. As such, for the present study, we test the following hypotheses:

**H1:**  *Students in the two-year Scholars program will report higher levels of research skills at the end of the program than students in the one-year Fellows Program.*

**H2:**  *Scholars will report higher levels of science identity, researcher identity, belongingness to the program and profession, cultural compatibility, and time management than Fellows.*

**H3:**  *Scholars will show greater research productivity (i.e., more presentations and publications) than Fellows.*

## 2. Measures and Methods

The methods used in the present study are consistent with a protocol that was approved by the Institutional Review Board (IRB) at California State University, Long Beach.

### 2.1. Participants

There were 117 BULD trainees from five cohort years (2019–2023; 54.7% Fellows, 45.2% Scholars) included in this study. Trainees by cohort year are displayed in Table 2. For this study, the term cohort year is defined as a group of trainees with the same program entry year. Since Scholars spent two academic years in the program, they completed the training in the year following their specified cohort year.

Demographics of BUILD trainees (see Table 3) indicated there were more women (65.8%) than men. Almost half of trainees identified as Hispanic/Latino (46.2%), while other trainees identified as Asian/Asian American (24.8%), White (18.8%), Black/African American (5.1%), or two or more races/ethnicities (5.1%). Almost two-thirds of trainees were from lower financial backgrounds (i.e., Pell-eligible; 61.5%), and a third (33.3%) were first-generation college students. Before joining, 61.5% of trainees had previous research experience (*N* = 72/117).

The students in the Scholars and Fellows programs were similar in terms of gender (*p* = 0.963), race/ethnicity (*p* = 0.961), Pell-eligibility (*p* = 0.597), age (*p* = 0.054), and first-generation status (*p* = 0.511). Students in both programs had similar previous research prior to BUILD (*p* = 0.196). There was a small statistically significant difference in transfer status by program (*p* = 0.014, *Φ* = 0.228): Fellows were more likely to be transfer students (42.2%) than Scholars (20.8%). There was a small significant difference in college by program, (*p* = 0.046, *Φ* = 0.250): Fellows were more likely to be health and human services majors (21.9%; e.g., health science, nutrition) and natural sciences and mathematics majors (26.6%; e.g., biological sciences, chemistry) than Scholars (7.5%, 18.9%; respectively), while Scholars were more likely engineering majors (32.1%) and liberal arts majors (41.5%; e.g., psychology) than Fellows (18.8%, 32.8%; respectively). These variables were included as control variables in all models.

### 2.2. Measures

#### 2.2.1. Psychosocial Measures

Psychosocial measures were collected for evaluation at the start of the program in summer (June) and the end of each academic year in spring (May) on a Likert-type scale (1 = strongly disagree, 6 = strongly agree). When possible, existing scales were used, although some items were created to measure key program objectives for annual evaluation. To decrease survey fatigue and to increase validity, some items from scales were excluded or adapted to tailor items to the program, which was open to students in both the behavioral and biomedical disciplines. All items are included in Appendix A, and Cronbach’s alphas were calculated. Items measured *perceived research skills* adapted from the 20-item SURE scale (Lopatto, 2004), α = 0.92 (e.g., “I have the ability to analyze data and other information”; 18 items, Cronbach’s α = 0.90), *science identity* (e.g., “I see myself as a scientist”; 3 items, Cronbach’s α = 0.75), *researcher identity* (e.g., “I see myself as a researcher”; 3 items, Cronbach’s α = 0.68), three types of *belongingness* (*CSULB belonging*, e.g., “I see myself as part of the CSULB community”, 3 items, Cronbach’s α = 0.86; Program belonging adapted from the Perceived Cohesion Scale [PCS; Bollen and Hoyle (1990)], Cronbach’s α = 0.84, e.g., “I feel a sense of belonging within the BUILD community”, 4 items, Cronbach’s α = 0.96; and Professional belonging, e.g., “I feel confident that I will ‘fit in’ in the professional community of my discipline”, 4 items, Cronbach’s α = 0.65), *cultural compatibility* (e.g., “My cultural background is compatible with my future career”; 10 items informed by Cultural Congruency Scale, [CSS; Gloria and Robinson Kurpius (1996)], Cronbach’s α = 0.83, *family support* (e.g., “My family fully supports my educational goals”; 8 items, Cronbach’s α = 0.72). Five items measured *personal resource management skills* adapted from Neill (2016), Cronbach’s α = 0.79 (e.g., “I am able to meet deadlines;” 1 = I cannot do this at all, 5 = I can do this extremely well, midpoint 3 = I can do this a moderate amount, Cronbach’s α = 0.79). Items were reverse-coded when needed (see Appendix A). Scores were averaged to create composite scores for each construct with pre- and post-scores (1 year) for Fellows and pre- and post-scores (year 2) for Scholars. Relationships between all composite variables are also available in Appendix A (see Table A2).

The following measures were taken to reduce social desirability for all participants. First, students were informed that data were collected by external evaluators rather than program staff or faculty. Second, data were collected confidentially via survey links. Students were informed that their data remained confidential from the program (data were not linked with names or student IDs). Third, demographics were not collected alongside survey responses. Fourth, students were able to skip items or indicate responses like “Not Applicable”. Lastly, reminders were added to blocks of questions that were sensitive in nature, reminding trainees that student responses varied widely, and that there were no “right” or “wrong” responses. In addition, the participants were reminded of the confidential nature of their response. Although we included these methods to reduce the possibility of social desirability, we acknowledge that social desirability cannot be completely eliminated.

#### 2.2.2. Presentations, Awards, and Publications

The training program collected data from the following sources: applications, follow-up surveys, and curriculum vitae (CVs). Counts were created for each type of achievement metric for each trainee’s first year and the Scholars’ second year. For Fellows, data prior to joining BUILD (junior year) was also collected from CVs and training program applications.

- Presentations: A total of presentation counts was disaggregated into student (e.g., campus-based, such as the University Student Research Competition) and professional conference counts.
- Awards: Awards (e.g., travel awards, departmental awards) were summed for each trainee.
- Publications: Total publications included both peer-reviewed journal articles and published peer-reviewed conference proceedings.

#### 2.2.3. Educational Achievement

Cumulative GPA and bachelor’s degree attainment were obtained from the university’s Institutional Research (IR) office.

#### 2.2.4. Graduate School

Graduate school enrollment was collected from the National Student Clearinghouse (NSC), which is a standardized database for colleges and universities in the United States, through the university’s IR office in early October of 2025. Enrollment was recorded for each trainee and further for both master’s and doctoral programs. Based on enrollment codes from NSC, doctoral programs were further disaggregated into PhD and non-PhD doctoral programs.

#### 2.2.5. Program and Demographics

The program was binary coded (0 = Scholars, 1 = Fellows). Demographics were obtained by IR. Previous research experience was collected by evaluators via survey at the start of BUILD and when missing, supplemented by program data.

#### 2.2.6. Discipline

Students from the Colleges of Health and Human Services (e.g., health sciences) and the College of Liberal Arts (e.g., psychology) were coded as behavioral science majors, and those from the College of Engineering (e.g., biomedical engineering) and Natural Sciences and Mathematics (e.g., biology) were coded as biomedical sciences.

#### 2.2.7. Statistical Analysis

Analyses were conducted using IBM SPSS (v. 29). Generalized linear models and logistic regression models examined differences in non-continuous outcomes by the BUILD program with demographics and previous research experience as controls. For continuous psychosocial outcomes, Mplus (v.8) was used to analyze path analysis models with robust standard errors. Path analysis models were employed to examine BUILD program cohort relationships with outcomes while also controlling for covariates and including correlations between some outcomes multivariately (see Appendix A, Table A2 for construct correlations). Five models were estimated:

1. A multivariate research outcome model: Perceived competence of research skills, science identity, and research identity outcomes.
2. A multivariate belongingness outcome model: CSULB Belongingness and BUILD Belongingness outcomes.
3. A multivariate Professional outcomes model: Professional Belonging and Cultural Career Congruency outcomes.
4. A univariate Resource Management model.
5. A univariate Family Support model.

Which outcome variables to include in each model was decided by considering conceptual overlap and composite correlations. This approach kept models relatively simple due to a smaller sample size while also modeling important outcome covariances.

Although all trainees were invited to participate in the spring evaluation survey for the psychosocial items, it was more common that early Fall Semester graduates did not respond. Participants were also allowed to skip items or select *I am not sure/I do not know* type responses, which were coded as missing. Missing data ranged from 7 to 20%. To avoid bias and loss of power if listwise deletion was employed, multiple imputations with chained equations with auxiliary variables were employed (Enders, 2022). Control variables, including pre-measures of constructs, were included in the imputation models as auxiliary variables because missingness was related to covariates (MAR). Graduating early (i.e., Fall) was more typical for Scholars (*n* = 9, 17%) than Fellows (*n* = 4, 6.3%). Therefore, covariates in the imputation model included the BUILD program cohort membership and demographics, and all pre-measures were included in the model for missing data estimation. A total of 100 datasets were imputed, and the results of statistical tests were pooled across the datasets and reported for psychosocial constructs. All estimates in the imputed datasets were within the boundaries of possible scores.

## 3. Results

### 3.1. Psychosocial Constructs

At the conclusion of their participation in the BUILD program, students reported high perceived research skills, science identity, and research identity (*M*s = 4.89–5.21), see Table 4. Belongingness to the BUILD program and CSULB were also quite high (*M*s = 5.13), while family support, cultural compatibility, and time management were slightly lower and more mixed (*M*s = 3.82–4.52), with averages between somewhat disagree/somewhat agree. Overall, trainees also reported lower professional belonging (*M* = 3.76) compared to their belongingness to the BUILD program and the university. Psychosocial constructs by the BUILD program are also presented univariately below without control variables first (Table 4), followed by results of statistical models with control variables (Table 5 and Table 6).

When examining differences in psychosocial outcomes by the BUILD program, including covariates, path analysis models were analyzed with the BUILD program as a binary predictor so that differences between the programs were estimated while controlling for both the psychosocial variables at the start of the program (pre-measures) and demographics. As a reminder, pooled results are presented because a multiple imputation method was employed to handle missing data (see Section 2). The first research-related psychosocial outcomes results in Table 5 indicated that Fellows had lower perceptions of their research skills compared to Scholars at the end of the program, *b* = −0.28, *p* < 0.05, controlling for demographics and pre-measures of the three outcomes. However, Fellows and Scholars had similar post-science (*b* = −0.17, *p* = 0.253) and post-research identity (*b* = −0.16, *p* = 0.221). Pre-research skills were positively related to post-research skills (*p* < 0.001) and post-researcher identity (*p* = 0.018) but not science identity (*p* = 0.530). Pre-science identity was not related to any of the post outcomes (*p*s = 0.287–0.548). Pre-researcher identity was positively related to post-researcher identity (*p* = 0.005), research skills (*p* = 0.022) and science identity (*p* = 0.008). 

All other differences in the remaining psychosocial outcomes between the BUILD Cohort Program (Fellow vs. Scholar) were non-significant (see Table 6, Table 7, Table 8 and Table 9). For belongingness outcomes, there were no differences between Fellows and Scholars (BUILD Cohort) at the end of BUILD for either CSULB (*p* = 0.393) or BUILD belongingness (*p* = 0.229), controlling for covariates, see Table 6. Pre-CSULB belongingness was related to both post-CSULB belongingness (*p* < 0.001) and BUILD belongingness (*p* = 0.013).

For Professional outcomes, there were no differences between Fellows and Scholars (BUILD Cohort) at the end of BUILD in terms of cultural compatibility (*p* = 0.136) or professional belongingness (*p* = 0.468), controlling for covariates, see Table 7. Pre-cultural compatibility and professional belonging were positively related to their respective post outcomes, *p*s < 0.001, see Table 7.

For personal resource management, there were no differences between Fellows and Scholars (BUILD Cohort) at the end of BUILD, controlling for covariates (see Table 8, *p* = 0.136). Pre-personal resource management was positively related to the post measurement, *p* < 0.001.

For family support, there was no difference between Fellows and Scholars (BUILD Cohort) at the end of BUILD, controlling for covariates (see Table 9, *p* = 0.050). Pre-family support was positively related to post-family support, *p* < 0.001.

### 3.2. Presentations

Descriptive statistics for average presentations by type of presentation, year, and BUILD program are displayed in Table 10. On average, BUILD trainees had 4–5 student and/or professional conference presentations during their junior and senior years (*M* = 4.49, *Md* = 4.00, *SD* = 2.83, *Min* = 0, *Max* = 17).

Poisson models of counts of presentations indicated that, controlling for demographics and previous research experience, Fellows had fewer total, professional, and student presentations across both their junior and senior years combined compared to Scholars by the end of their BUILD program (see Table 11).

Next, we examined outcomes by time periods with the same control variables. During junior year, Scholars had more presentations regardless of type than Fellows, who were not yet in BUILD but many of whom were already engaged in research (*p*s < 0.001). When Scholars completed their second year of BUILD and Fellows their first year, the difference in the average presentations between the two groups during senior year was non-significant (*p* = 0.448). However, the effect of the BUILD program on presentations depended on the type of presentation, such that Scholars had more professional presentations than Fellows by the end of their senior year (*p* = 0.047), while Fellows had more student presentations than Scholars (*p* < 0.001).

### 3.3. Publications

While most BUILD trainees did not publish their BUILD-sponsored research (*N* = 90, 76.9%), some trainees published their research once (11%, *N* = 13), 3.4% published twice (*N* = 4), and one trainee published three times (0.9%) across their junior and senior years. Nine students did not have data recording publications (7.7%). Since the number of publications was low, we analyzed BUILD program differences in whether a trainee was published or not, regardless of the number of publications. There was a statistically significant association between publishing and the BUILD program, *X*^2^(1) = 5.12, *p* = 0.024, *Φ* = 0.22 (a small-moderate effect), such that 26.1% of Scholars published research (*N* = 12), while 9.7% of Fellows published research (*N* = 6). The BUILD program difference in publishing was statistically significant when controlling for demographics and prior research experience using logistic regression. The odds of Fellows publishing were 0.18 times the odds of Scholars, ∆*X*^2^ (1) = 5.42, *p* = 0.020, *b* = −1.48, *SE* = 0.67, *OR* = 0.18, *R*^2^*_N_* = 0.27, indicating lower odds.

### 3.4. Awards

Although half of trainees indicated that they did not earn an award (50.4%, *N* = 59), about a third of trainees earned one award (28.2%, *N* = 33), 12.8% earned two awards (*N* = 15), 4.3% earned three (*N* = 5), 2.6% earned four (*N* = 3), 0.9% earned six awards (*N* = 1), and 0.9% earned 14 awards (*N* = 1). Differences in earning at least one award versus not earning an award between BUILD programs were tested with logistic regression models controlling for demographics and previous research experience. In total, across both junior and senior years, 52.8% of Scholars earned at least one award (*N* = 28), and 46.9% of Fellows earned at least one award (*N* = 30). The BUILD program difference was non-significant, *b* = −0.10, *SE* = 0.42, ∆*X*^2^(1) = 0.06, *p* = 0.806. For awards earned across junior year, proportions were similar, such that 22.6% of Scholars earned an award (*N* = 12) during their first year in BUILD, while 17.2% of Fellows earned an award (*N* = 11) while not yet in BUILD. The BUILD program difference was non-significant, *b* = 0.20, *SE* = 0.53, ∆*X*^2^(1) = 0.14, *p* = 0.706. For awards earned across senior year when both groups were in BUILD, proportions were similar, such that 39.6% of Scholars earned an award (*N* = 21) and 34.4% of Fellows earned an award (*N* = 22). The BUILD program difference was also non-significant, *b* = −0.40, *SE* = 0.46, ∆*X*^2^(1) = 0.77, *p* = 0.379.

Poisson models of total counts of awards across both junior and senior years indicated that there were no statistically significant differences by BUILD program, controlling for demographics and previous research experience (*p*s = 0.254–0.402, see Table 12). Zero-Inflated Poisson models also indicated that the BUILD Program predictor was non-significant for total awards, senior year and junior year for both the zero-inflation (*p*s = 0.098, 0.080, 0.061; respectively) and count portions of the models (*p*s = 0.690, 0.083, 0.669; respectively).

### 3.5. Doctoral Enrollments

There were 37 doctoral enrollments of 117 students for this study (31.6%). For Fellows, 35.9% (*N* = 23/64) enrolled in a doctoral program, while for Scholars, 26.4% enrolled (*N* = 14/53), see Table 13.

There was no difference in the rate of enrollment in any doctoral degree program by the BUILD program with controls, ∆*X*^2^(1) = 1.43, *p* = 0.267, *b* = 0.57, *SE* = 0.45 (see Table 14).

There were 34 BUILD trainees who enrolled in PhD programs. There were no differences in the rate of enrollment in the PhD degree program by the BUILD program ∆*Χ*^2^(1) = 0.97, *p* = 0.325, *b* = 0.51, *SE* = 0.46.

### 3.6. Master’s Degree

A little over a third (35.9%) of BUILD trainees enrolled in a Master’s granting program (*N* = 42), see Table 15.

Fellows enrolled in master’s degree programs (*N* = 24/64, 37.5%) at a similar rate as Scholars (*N* = 18/53, 34.0%). When considering controls using logistic regression, there was no difference in the rate of enrollment in the master’s degree program by BUILD program, ∆*X*^2^(1) = 0.16, *p* = 0.689, *b* = 0.28, *SE* = 0.43, see Table 16.

Of all trainees, 20.5% enrolled in a master’s degree program and earned a master’s degree (*N* = 24/117) thus far. Of the 42 enrolled in a master’s program, 24 earned a master’s degree (*N* = 24/42; 57.1%) thus far. Among these, fewer Scholars (15%, *N* = 8) than Fellows (25%, *N* = 16) earned master’s degrees thus far. When including controls into the model, there were no differences in earning master’s degrees by the BUILD Program, ∆*X*^2^(1) = 1.78, *p* = 0.182, *b* = 0.85, *SE* = 0.57.

### 3.7. Cumulative GPA

The average cumulative GPA of trainees at the time of degree was 3.65 (*SD* = 0.29, *Min* = 2.73, *Max* = 4.00). GPAs were negatively skewed, with 50% of the scores at or above 3.71. Scholars’ average GPA was 3.66 (*SD* = 0.32, *Min* = 2.73, *Max* = 4.00, *Med* = 3.76) while Fellows’ was 3.64 (*SD* = 0.26, *Min* = 2.96, *Max* = 4.00, *Med* = 3.67). There was no difference in average cumulative GPA at degree time by BUILD program with control variables, *b* = −0.01, *SE* = 0.05, *t*(107) = −0.20, *p* = 0.845, see Table 17.

## 4. Discussion

Prior studies have investigated the impact of student participation in undergraduate research training programs on student success metrics (Arruda et al., 2025; Hall et al., 2016; Vu et al., 2023). A two-year program has been the standard for formal research training programs (e.g., NIH’s MARC legacy programs), highlighting the need for trainee growth over time. However, one study (Chamely-Wiik et al., 2023) found that beneficial student outcomes are achieved with three semesters of research participation (or two semesters plus a summer research experience). Allowing flexibility in the length of a research training program while maintaining its effectiveness will allow organizations to better accommodate students’ needs and available resources. One motivation for introducing flexibility in the second phase of the CSULB BUILD program was to provide different entry points for students, including first-generation and transfer students, as well as those in certain majors, particularly those in Health and Human Services majors. During the first phase of BUILD, we observed that students in these groups often became interested in research later in their academic careers, shifting from their original plans to pursue alternative career paths.

The goal of the present study was to examine differences in duration of research training by comparing a Scholars program modeled after NIH’s gold standard (i.e., 2-year research program) with an accelerated Fellows Program (summer, plus academic year) program. We compared the two programs on a variety of outcomes that were hallmarks of student success identified by NIH, such as the number of presentations and publications, the number of awards, GPA at graduation, and matriculation in graduate school, using NSC data. In addition, we compared psychosocial factors for the trainees, such as sense of belonging and research identity, across the two programs.

Contrary to predictions, we found that the one-year Fellows Program produced outcomes similar to the two-year Scholars program on most psychosocial variables, cumulative GPA, awards, and graduate enrollment. In terms of GPA at graduation and number of awards, there was no difference between outcomes achieved by Scholars and Fellows. Most of the awards obtained by students occurred during their last year for both program trainees, which reflects the fact that many awards are given during or aligned with commencement. The graduate school matriculation rates also did not differ between programs, suggesting that both the Scholars and the Fellows programs provided comparable support for graduate school preparation.

However, there were some benefits to participating in a program that is longer in duration. Scholars reported higher levels of research skills than Fellows at the end of the program, supporting H1, and had a higher number of publications and professional presentations, partially supporting H3. Thus, better outcomes relating to research productivity can be achieved in programs that are of longer duration because there are more opportunities to benefit from in the program over time. That is, Scholars had more time to engage in research across their junior and senior years. Scholars had more opportunities to develop their research projects for publication and professional presentations than their one-year counterparts. Overall, longer duration of research experience may provide greater opportunities for durable skill development, through greater distributed practice, repeated iteration, cumulative gain in efficacy, and exposure to more professional experiences that unfold over time (e.g., annual conference cycles) in communities of practice.

Fellows had more student-focused presentations during senior year compared to Scholars, which could have been largely driven by BUILD program support and requirements. The BUILD program routinely took trainees to the student-centric conferences (e.g., Annual Biomedical Research Conference for Minoritized Scientists; Society for Advancement of Chicanos/Hispanics and Native Americans in Science) in their first year in the program, where students were strongly encouraged to present their research. This provided students the opportunity to travel as a group to (typically) their first conference in a student-friendly, culturally inclusive setting and was chaperoned by program faculty/staff. For Scholars, they had financial support during their second year in the program to present at a professional conference in their field, while Fellows had to choose between attending the student-focused conference with their peers or attending a professional conference with their mentor or on their own.

Contrary to the prediction that longer engagement would produce stronger identities and research/professional development (Adedokun et al., 2014), we did not find Fellows and Scholars to differ significantly on science identity, researcher identity, BUILD belonging, CSULB belonging, professional belonging, cultural compatibility, family support, or time management once covariates were included. The lack of a difference can be attributed to the shared features across the parallel cohort of Scholars and Fellows Program. Both programs were comparable in providing faculty-mentored research, structured Learning Community participation, opportunities for professional development and conference participation, and the inclusion of family events that highlighted the trainees’ achievements. This was also likely impacted by program admission criteria for Scholars and Fellows. Scholars could have entered the program without prior research experience, while Fellows were selected partly based on having already participated in some sort of research experience prior to applying. Thus, prior research experience may have been sufficient to initiate the development of students’ research identity.

We also found that both Scholars and Fellows reported high levels of belonging to BUILD and CSULB, but lower ratings for professional belonging (with Fellows showing slightly but not significantly lower levels than Scholars). This is understandable given the relatively short time these students have spent conducting research, and it will likely increase as students move on to pursue graduate degrees. Many of the BUILD students express strong ties to their families and local communities. This is one of the reasons the BUILD program encourages students to apply outside of Southern California for their summer research experience, so that they can learn what living away from family is like to better inform their graduate program application process. These findings are consistent with the notion that feeling connected to the broader research profession and integrating scientific aspirations with family and cultural identity may take longer and may require more explicit support than developing belonging to a specific campus program.

We note that a small number of our Scholars and Fellows graduated earlier than standard program length, but these students still accomplished all of the program goals. This suggests that duration itself is not the primary determinant of success. Rather, what is essential is an adequate duration of period of engagement that enables students to meet program objectives. Thus, we recommend offering both types of programs with added flexibility to meet the students’ varying timelines as they consider graduate school and become ready to commit to an intensive undergraduate research training program. Flexibility in the duration of the program will also allow organizations to maximize program resources by supporting some students for shorter periods of time, opening up remaining funding to support additional students.

One parameter we did not investigate in this study was whether longer duration preparation in undergraduate studies fosters improved graduate program and career outcomes. Historically, minoritized and first-generation students have lower representation in doctoral degree attainment and subsequent careers in the biomedical workforce (National Academy of Sciences, 2005). The BUILD program was created by the NIH specifically to address this diversity gap in the biomedical field. Logically, many of the metrics assessed in the current study are relatively short-term and focused on the key (but early) step of transitioning from undergraduate degrees into graduate studies in relevant biomedical and behavioral fields, but it would be instructive for future research studies to examine the impacts that the duration of enhanced early preparation has on long-term outcomes.

Limitations of this study include its correlational nature, preventing the ability to make strong causal claims, as this study lacked random assignment. We also acknowledge that selection bias is a limitation given the non-experimental design. Models included a set of relevant control variables (previous research experience, transfer status, baseline measures, and demographic characteristics); however, it is possible that other unmeasured covariates could be related to program participation and study outcomes. As is typical for undergraduate research programs that can only support a limited number of students, the sample size for this study was just over 100 students, which meant smaller effects may have gone undetected (Type II error). A larger sample size would allow for more complex relationships to be tested (e.g., interactions, mediation models), encouraged for future research studies. Finally, although students spanned both biomedical and behavioral disciplines, given that the students were from only one R2 university, external validity is reduced.

## 5. Conclusions

We found that Scholars, who enrolled in a 2-year research training program, had significantly higher ratings of research skills, number of publications, and number of professional presentations than Fellows, who enrolled in a 1-year accelerated research training program. However, Scholars and Fellows did not differ significantly with respect to GPA at graduation, number of awards or graduate school enrollment. In addition, trainees from the two programs did not differ on self-reported levels of science identity, researcher identity, belongingness, cultural compatibility, family support, or time management. These findings indicate that a compressed, shorter, 1-year program duration that is comparable in intensity could produce many of the broader social and academic benefits of a 2-year program that is considered the gold standard (Hall et al., 2016). However, programs that are longer in duration allow time for students to gain extended research experience and generate research publications and professional presentations. Thus, both types of programs should be available to best meet students’ needs and to make the most effective use of program resources.

## Figures and Tables

**Table 1:** Program elements of the CSULB BUILD (2019–2025) Scholars and Fellows Program.

Program Element	BUILD Scholars	BUILD Fellows
Primary Target Group	Students in a two-year sequence (commonly juniors who progressed to seniors and committed to doctoral study).	Seniors with some prior research experience and commitment to doctoral study.
Program Length	Two years ^1^ (Scholars 1: junior year → Scholars 2: senior year).	One year ^1^ (senior year).
Initial Summer Training	Mandatory 8-week summer program at entry plus intensive summer research with faculty mentor.	
Academic-year LC Course	Weekly Learning Community (1 unit). Meeting for 50 min once a week	
Additional Summer Research Experience	Encouraged external Summer Research Experience at R1 or home institution between first and second year	Not applicable
Academic-year Research Time	Substantial weekly faculty-mentored research commitment (~15 h/week).	
Student Financial Support	Included comparable financial support, but in a different format due to funding sources (stipend + tuition support for Scholars and bi-weekly salary support for Fellows).	
Mentor Supply Funds	Included, but at slightly higher levels for Scholars than Fellows due to the longer duration of training for Scholars	
Graduate School Preparation Emphasis	Emphasized for both Scholars and Fellows	
Family Support	Families were invited to participate in the BUILD Summer Research Symposium (where students present posters to families, mentors, and peers) and the BUILD Commencement at the end of the program	

^1^ Some Scholars graduated early in three semesters, while some Fellows graduated early after one semester, but were included in our analyses.

**Table 2:** Total unique participants by year of entry into the BUILD Program.

Cohort Year	Fellows		Scholars		Total	
	*n*	%	*n*	%	*N*	%
2019	15	23.4	14	26.4	29	24.8
2020	15	23.4	11	20.8	26	22.2
2021	11	17.2	8	15.1	19	16.2
2022	13	20.3	10	18.9	23	19.7
2023	10	15.6	10	18.9	20	17.1
Total	64	54.7	53	45.2	117	100

*Note*: One Scholar who joined in 2023 was counted as a Fellow due to graduating a full year early. One cohort 2020 Scholar separated late, but finished the program; therefore, was included as a completer. A Fellow went on leave in 2019 but rejoined in 2020 and, therefore, was included in the 2020 cohort instead of 2019.

**Table 3:** Demographics by program.

Demographic	Fellows (*n* = 64)		Scholars (*n* = 53)		Total (*N* = 117)	
	*n*	%	*n*	%	*N*	%
*Women*	42	65.6	35	66.0	77	65.8
*Race/Ethnicity*						
Hispanic/Latinx	30	46.9	24	45.3	54	46.2
Asian/Asian American	16	25.0	13	24.5	29	24.8
White	11	17.2	11	20.8	22	18.8
Black/African American	4	6.3	2	3.8	6	5.1
Two or more races	3	4.7	3	5.7	6	5.1
*Pell Eligible*	38	59.4	34	64.2	72	61.5
*First Generation*	23	35.9	16	30.2	39	33.3
*Transfer Student*	27	42.2	11	20.8	38	32.5
*College (Discipline)*						
Health and Human Services	14	21.9	4	7.5	18	15.4
Liberal Arts	21	32.8	22	41.5	43	36.8
Natural Sciences & Mathematics	17	26.6	10	18.9	27	23.1
Engineering	12	18.8	17	32.1	29	24.8
*Age at CSULB entry*	*M* = 20.03 (*SD* = 3.45)	17–32	*M* = 18.91 (*SD* = 2.66)	17–29	*M* = 19.52 (*SD* = 3.15)	17–32

*Note*: The minimum and maximum values are given for age rather than percentage.

**Table 4:** Pooled descriptive statistics and independent t-test results for psychosocial constructs by BUILD Program (*N* = 117, 100 Imputations).

Psychosocial Construct (Pooled Mean)	BUILD Program	Mean	*Mean*	*SE*	*t*	*df*	*p*
			*Diff*				
Research Skills (*M* = 5.21)	Fellows	5.10	−0.24	0.11	−2.21	1572	0.027
	Scholars	5.34					
Science Identity (*M* = 4.89)	Fellows	4.84	−0.11	0.19	−0.58	1814	0.566
	Scholars	4.95					
Research Identity (*M* = 5.07)	Fellows	4.98	−0.17	0.19	−0.89	1672	0.373
	Scholars	5.16					
CSULB Belonging (*M* = 5.13)	Fellows	5.12	−0.02	0.19	−0.08	2249	0.935
	Scholars	5.14					
Cultural Compatibility (*M* = 3.82)	Fellows	3.70	−0.26	0.20	−1.30	1007	0.196
	Scholars	3.96					
Professional Belonging (*M* = 3.76)	Fellows	3.69	−0.14	0.20	−0.74	2039	0.461
	Scholars	3.83					
Family Support (*M* = 4.52)	Fellows	4.45	−0.15	0.16	−0.97	2253	0.332
	Scholars	4.61					
Time Management (*M* = 4.01)	Fellows	4.00	−0.02	0.17	−0.13	1254	0.897
	Scholars	4.02					
BUILD Belonging (*M* = 5.13)	Fellows	5.07	−0.13	0.26	−0.51	1292	0.614
	Scholars	5.20					

*Note*: Fellows (*N* = 64), Scholars (*N* = 53), equal variances were not assumed, pooled degrees of freedom (*df*s) were based on Rubin’s Rules, Cohen’s *d*s for Research Skills ranged 0.25 to 0.74.

**Table 5:** Research outcomes at the end of BUILD regressed on BUILD Cohort Program and control variables (*N* = 117).

	Research-Related Psychosocial Outcomes at End of BUILD											
Predictor	Post-Research Skills (*R*^2^ = 0.46)				Post-Science Identity (*R*^2^ = 0.36)				Post-Researcher Identity (*R*^2^ = 0.43)			
	Est.	SE	Est/SE	*p*	Est.	SE	Est/SE	*p*	Est.	SE	Est/SE	*p*
BUILD Cohort (1 = Fellow)	−0.28	0.08	−3.61	<0.001	−0.17	0.14	−1.14	0.253	−0.16	0.13	−1.23	0.221
Pre Research Skills	0.34	0.08	4.16	<0.001	0.09	0.15	0.63	0.530	0.30	0.13	2.37	0.018
Pre Science Identity	−0.09	0.09	−1.07	0.287	0.13	0.16	0.82	0.411	0.08	0.13	0.60	0.548
Pre Researcher Identity	0.21	0.09	2.30	0.022	0.41	0.16	2.63	0.008	0.37	0.13	2.82	0.005
Transfer Student	0.04	0.08	0.47	0.639	0.13	0.17	0.77	0.442	−0.24	0.14	−1.72	0.086
Women	−0.10	0.09	−1.14	0.254	−0.09	0.17	−0.50	0.615	0.35	0.16	2.16	0.031
First Generation	−0.09	0.10	−0.98	0.327	−0.27	0.15	−1.82	0.069	0.06	0.14	0.39	0.694
Pell Eligible	−0.06	0.09	−0.68	0.494	−0.18	0.16	−1.16	0.248	0.05	0.14	0.36	0.719
Program Year (Centered)	0.09	0.03	3.40	0.001	0.10	0.05	2.07	0.038	0.05	0.05	1.09	0.275
Prev. Research Experience (1 = Yes)	0.08	0.08	0.98	0.325	−0.05	0.15	−0.36	0.722	−0.06	0.13	−0.44	0.664
Discipline (1 = Behavioral)	0.00	0.09	0.00	0.998	0.08	0.17	0.49	0.625	−0.05	0.16	−0.33	0.740
Asian	−0.18	0.10	−1.72	0.085	−0.35	0.17	−2.03	0.042	−0.20	0.16	−1.23	0.217
White	−0.03	0.11	−0.30	0.764	−0.42	0.18	−2.31	0.021	−0.37	0.16	−2.29	0.022
Black/Other	0.00	0.13	0.00	0.998	−0.42	0.24	−1.71	0.087	−0.41	0.27	−1.55	0.122
Residual Covariances (Correlations) of Outcomes	**Post-Research Skills**				**Post-Science Identity**				**Post-Researcher Identity**			
	Cov (Corr)	SE	Est/SE	*p*	Cov (Corr)	SE	Est/SE	*p*	Cov (Corr)	SE	Est/SE	*p*
Post-Researcher Identity	0.13(1.00)	0.02	7.77	<0.001								
Post-Science Identity	0.12 (0.52)	0.03	4.33	<0.001	0.42(1.00)	0.07	5.90	<0.001				
Post-Researcher Identity	0.11 (0.50)	0.03	4.31	<0.001	0.25 (0.63)	0.06	3.86	<0.001	0.37(1.00)	0.07	5.12	<0.001

*Note.* Pooled estimates based on 100 imputations, race/ethnicity referent is Latinx/Hispanic and for discipline, biomedical. None of the pre-measures were related to the BUILD program cohort (*p*s = 0.514–0.973). Cov = covariance, Corr = correlation.

**Table 6:** Belonging outcomes at the end of BUILD regressed on BUILD Cohort Program and control variables (*N* = 117).

	Belonging Related Psychosocial Outcomes at End of BUILD							
Predictor	CSULB Belonging (*R*^2^ = 0.32)				BUILD Belonging (*R*^2^ = 0.14)			
	Est.	SE	Est/SE	*p*	Est.	SE	Est/SE	*p*
BUILD Cohort (1 = Fellow)	−0.14	0.17	−0.85	0.393	−0.26	0.22	−1.20	0.229
CSULB Belonging (pre)	0.43	0.08	5.49	<0.001	0.31	0.12	2.48	0.013
Transfer Student	−0.11	0.21	−0.53	0.596	−0.06	0.25	−0.24	0.809
Women	0.20	0.18	1.14	0.256	0.04	0.23	0.20	0.845
First Generation	0.18	0.15	1.26	0.208	0.04	0.22	0.20	0.839
Pell Eligible	−0.05	0.19	−0.26	0.795	0.12	0.22	0.53	0.594
Program Year (Centered)	0.05	0.05	1.03	0.303	−0.02	0.08	−0.29	0.775
Prev. Research Experience	−0.26	0.15	−1.74	0.082	−0.32	0.19	−1.66	0.097
Discipline (1 = Behavioral)	−0.08	0.15	−0.56	0.576	0.08	0.21	0.39	0.697
Asian	0.03	0.17	0.16	0.877	0.02	0.27	0.07	0.941
White	−0.21	0.20	−1.04	0.298	−0.30	0.29	−1.05	0.294
Black/Other	0.13	0.24	0.55	0.584	0.29	0.27	1.06	0.289
**Residual Covariances** **(Correlations)**	**Cov (Corr)**	**SE**	**Est/SE**	***p***	**Cov (Corr)**	**SE**	**Est/SE**	***p***
CSULB Belonging	0.44 (1.00)	0.10	4.36	<0.001				
BUILD Belonging	0.34 (0.53)	0.09	3.62	<0.001	0.90 (1.00)	0.21	4.30	<0.001

*Note.* Pooled unstandardized estimates based on 100 imputations, race/ethnicity referent is Latinx/Hispanic and for discipline, biomedical. The CSULB pre-measure was not related to the BUILD program cohort membership (*p* = 0.339). No pre-measurement for BUILD Belonging as trainees hadn’t started yet.

**Table 7:** Professional outcomes at the end of BUILD regressed on BUILD Cohort Program and control variables (*N* = 117).

	Professional Psychosocial Outcomes at End of BUILD							
Predictor	Post Cultural Compatibility (*R*^2^ = 0.38)				Post Professional Belonging (*R*^2^ = 0.33)			
	Est.	SE	Est/SE	*p*	Est.	SE	Est/SE	*p*
BUILD Cohort (1 = Fellow)	−0.23	0.15	−1.49	0.136	−0.11	0.16	−0.73	0.468
Cultural Compatibility (Pre)	0.33	0.09	3.85	<0.001	0.05	0.10	0.51	0.609
Professional Belonging (Pre)	0.15	0.08	1.88	0.061	0.35	0.09	4.05	<0.001
Transfer Student	−0.04	0.18	−0.22	0.826	−0.07	0.20	−0.37	0.712
Women	0.33	0.16	2.07	0.038	0.34	0.17	1.99	0.047
First Generation	−0.13	0.16	−0.82	0.415	0.13	0.18	0.72	0.469
Pell Eligible	−0.05	0.14	−0.38	0.708	0.06	0.16	0.37	0.709
Program Year (Centered)	0.11	0.04	2.61	0.009	0.10	0.05	1.97	0.049
Prev. Research Experience	−0.05	0.13	−0.37	0.713	0.07	0.15	0.46	0.644
Discipline (1 = Behavioral)	0.09	0.14	0.68	0.494	0.10	0.14	0.68	0.494
Asian	−0.29	0.17	−1.70	0.090	−0.35	0.19	−1.86	0.062
White	−0.33	0.18	−1.86	0.063	−0.18	0.20	−0.91	0.365
Black/Other	−0.05	0.18	−0.28	0.778	0.05	0.20	0.27	0.788
**Residual Covariances** **(Correlations)**	**Cov** **(Corr)**	**SE**	**Est/SE**	***p***	**Cov (Corr)**	**SE**	**Est/SE**	***p***
Cultural Compatibility	0.62 (1.00)	0.09	6.84	<0.001				
Professional Belonging	0.22 (0.51)	0.06	3.41	0.001	0.67(1.00)	0.09	7.17	<0.001

*Note*. Pooled unstandardized estimates based on 100 imputations, race/ethnicity referent is Latinx/Hispanic, and for discipline, biomedical. The pre-measures of the constructs were related, Cov. = 0.30 (Corr. = 0.34), SE = 0.09, *p* = 0.001 but not related to the BUILD program cohort (*p*s = 0.536, 0.668).

**Table 8:** Personal resource management at the end of BUILD regressed on BUILD Cohort Program and control variables (*N* = 117).

Predictor	Post Time Management *R*^2^ = 0.27			
	Est.	SE	Est/SE	*p*
BUILD Cohort (1 = Fellow)	−0.05	0.12	−0.37	0.712
Personal Resource Management (Pre)	0.46	0.09	4.86	<0.001
Transfer Student	−0.14	0.13	−1.07	0.285
Women	0.06	0.13	0.46	0.649
First Generation	−0.12	0.15	−0.79	0.431
Pell Eligible	−0.09	0.13	−0.69	0.491
Program Year (Centered)	0.06	0.04	1.65	0.098
Prev. Research Experience	−0.12	0.12	−0.96	0.337
Discipline (1 = Behavioral)	0.09	0.13	0.73	0.466
Asian	−0.18	0.15	−1.21	0.226
White	−0.05	0.19	−0.25	0.807
Black/Other	−0.25	0.18	−1.36	0.175

*Note*. Pooled unstandardized estimates based on 100 imputations, race/ethnicity referent is Latinx/Hispanic and for discipline, biomedical.

**Table 9:** Family support at the end of BUILD regressed on BUILD Cohort Program and control variables (*N* = 117).

Predictor	Post-Family Support *R*^2^ = 0.47			
	Est.	SE	Est/SE	*p*
BUILD Cohort (1 = Fellow)	−0.22	0.11	−1.96	0.050
Pre-Family	0.50	0.09	5.75	<0.001
Transfer Student	−0.15	0.13	−1.13	0.257
Women	−0.19	0.13	−1.48	0.138
First Generation	−0.15	0.13	−1.14	0.256
Pell Eligible	−0.13	0.12	−1.06	0.288
Program Year (Centered)	0.06	0.04	1.60	0.110
Prev. Research Experience	−0.02	0.11	−0.15	0.884
Discipline (1 = Behavioral)	0.03	0.11	0.29	0.775
Asian	0.05	0.13	0.37	0.709
White	−0.10	0.17	−0.60	0.550
Black/Other	−0.06	0.17	−0.34	0.733

*Note*. Pooled unstandardized estimates based on 100 imputations, race/ethnicity referent is Latinx/Hispanic and for discipline, biomedical.

**Table 10:** Presentations by type, student year, and BUILD Program (*N* = 117).

BUILD Program	Student Presentations			Professional Presentation			Total Presentations		
	Junior	Senior	Total	Junior	Senior	Total	Junior	Senior	Total
Fellows (*N* = 64)									
Mean	0.41	2.23	2.64	0.13	0.77	0.89	0.53	3.00	3.53
(SD)	(0.85)	(1.39)	(1.47)	(0.42)	(1.07)	(1.17)	(1.05)	(1.71)	(1.94)
Min, Max	0, 5	0, 6	0, 6	0, 2	0, 4	0, 4	0, 6	0, 8	0, 8
Scholars (*N* = 53)									
Mean	2.15	1.42	3.57	1.09	1.21	2.30	3.25	2.62	5.87
(SD)	(1.12)	(1.17)	(1.62)	(1.33)	(1.59)	(2.54)	(1.78)	(2.09)	(3.21)
Min, Max	0, 5	0, 5	1,8	0, 6	0, 6	0, 11	1, 9	0, 9	1, 17
BUILD Program (*N* = 117)									
Mean	1.20	1.86	3.06	0.56	0.97	1.53	1.76	2.83	4.59
(SD)	(1.31)	(1.35)	(1.60)	(1.06)	(1.34)	(2.03)	(1.96)	(1.89)	(2.83)
Min, Max	0, 5	0, 6	0, 8	0, 6	0, 6	0, 11	0, 9	0, 9	0, 17

*Note*: Fellows were not in the BUILD training program during their junior year.

**Table 11:** Poisson regression results of total, professional, and student presentations on the program with control variables (*N* = 117).

Predictors	Total Presentations				Professional Presentations				Student Presentations			
	*b*	*SE*	*X* ^2^	*p*	*b*	*SE*	*X* ^2^	*p*	*b*	*SE*	*X* ^2^	*p*
Intercept	1.35	0.20	46.06	<0.001	−0.39	0.40	0.97	0.325	1.18	0.16	51.75	<0.001
Fellow	−0.53	0.10	−29.67	<0.001	−1.02	0.21	23.00	<0.001	−0.30	0.09	11.29	<0.001
Transfer	0.09	0.09	0.89	0.345	0.34	0.22	2.41	0.121	0.00	0.10	0.00	0.971
Women	0.16	0.10	2.86	0.091	0.06	0.22	0.07	0.796	0.20	0.08	5.46	0.019
First Generation	0.05	0.12	0.16	0.693	0.05	0.24	0.05	0.832	0.05	0.11	0.19	0.661
Pell Eligible	0.03	0.12	0.05	0.822	0.13	0.24	0.30	0.582	−0.03	0.09	0.13	0.715
Program Year- Centered	−0.02	0.03	0.41	0.520	0.03	0.06	0.28	0.596	−0.04	0.03	2.37	0.124
Prior Research Experience (1 = Yes)	0.12	0.10	1.38	0.240	0.32	0.24	1.82	0.178	0.02	0.09	0.04	0.837
Discipline (1 = Behavioral)	0.18	0.09	3.94	0.047	1.10	0.19	32.74	<0.001	−0.23	0.09	6.95	0.008
Race/ethnicity-Asian	0.19	0.15	1.71	0.191	0.24	0.27	0.75	0.388	0.16	0.13	1.52	0.218
Race/ethnicity-White	0.05	0.13	0.14	0.706	0.28	0.26	1.18	0.278	−0.08	0.13	0.42	0.516
Race/ethnicity-Other	0.26	0.15	3.10	0.078	−0.10	0.36	0.08	0.784	0.37	0.11	10.62	0.001
Model Omnibus Test:	*X*^2^(11) = 48.48, *p* < 0.001				*X*^2^(11) = 87.06, *p* < 0.001				*X*^2^(11) = 19.80, *p* = 0.048			

*Note*: Referent group for race/ethnicity is Hispanic/Latinx, and for discipline, biomedical discipline.

**Table 12:** Poisson regression results of awards on program with control variables (*N* = 117).

Predictors	Total Awards				Senior Year Awards				Junior Year Awards			
	*b*	*SE*	*X* ^2^	*p*	*B*	*SE*	*X* ^2^	*p*	*b*	*SE*	*X* ^2^	*p*
Intercept	0.84	0.55	2.33	0.127	0.71	0.43	2.74	0.098	−0.10	1.40	0.01	0.946
Fellow	−0.37	0.32	1.30	0.254	−0.31	0.31	0.94	0.333	−0.49	0.58	0.70	0.402
Transfer	−0.30	0.34	0.75	0.387	0.03	0.38	0.01	0.928	−1.07	0.59	3.32	0.069
Women	−0.04	0.23	0.04	0.849	−0.31	0.27	1.35	0.245	0.49	0.45	1.18	0.277
First Generation	−0.91	0.32	8.17	0.004	−0.98	0.40	5.93	0.015	−0.78	0.52	2.28	0.131
Pell Eligible	−0.09	0.31	0.09	0.762	0.02	0.28	0.01	0.941	−0.24	0.58	0.17	0.682
Program Year- Centered	−0.08	0.07	1.19	0.276	−0.14	0.08	2.94	0.087	0.02	0.15	0.02	0.894
Prior Research Experience (1 = Yes)	−0.58	0.29	3.87	0.049	−0.52	0.28	3.36	0.067	−0.63	0.55	1.32	0.251
Discipline (1 = Behavioral)	0.28	0.26	1.15	0.283	0.47	0.28	2.82	0.093	0.01	0.53	0.00	0.984
Race/ethnicity-Asian	−0.29	0.35	0.67	0.412	−0.05	0.36	0.02	0.888	−0.77	0.69	1.26	0.262
Race/ethnicity-White	−0.32	0.37	0.76	0.382	−0.46	0.40	1.37	0.241	−0.14	0.61	0.05	0.816
Race/ethnicity-Other	−0.55	0.55	1.00	0.318	−0.63	0.62	1.03	0.310	−0.55	0.80	0.47	0.494
Model Omnibus Test:	*X*^2^(11) = 36.72, *p* < 0.001				*X^2^*(11) = 25.85, *p* = 0.007				*X*^2^(11) = 23.58, *p* = 0.015			

*Note*: The referent group for race/ethnicity is Hispanic/Latinx and biomedical for discipline.

**Table 13:** Doctoral enrollments by program and cohort year.

Cohort Year	Fellows (*n* = 64)	Scholars (*n* = 53)
2019	4	4
2020	5	4
2021	5	2
2022	4	2
2023	5	2
**Total**	**23/64 (35.9%)**	**14/53 (26.4%)**

*Note*: NSC data pulled 7 October 2025.

**Table 14:** Logistic regression results for doctoral program enrollment (*N* = 117).

Predictor	Doctoral Program Enrollment					
	*b*	S.E.	Wald	*df*	*p*	Exp(B)
BUILD Program (Fellow = 1)	0.57	0.45	1.64	1	0.200	1.77
Transfer Student	0.28	0.50	0.31	1	0.577	1.32
Women	0.35	0.49	0.50	1	0.480	1.41
First Generation	−0.92	0.55	2.79	1	0.095	0.40
Pell Eligible	0.05	0.49	0.01	1	0.926	1.05
Discipline-Behavioral	−0.85	0.48	3.21	1	0.073	0.43
Race/Ethnicity (Ref = Latine)			3.35	3	0.341	
Asian	−0.86	0.60	2.10	1	0.147	0.42
White	−1.02	0.65	2.46	1	0.116	0.36
Other Race/Ethnicity	−0.54	0.76	0.51	1	0.474	0.58
Program (Centered)	0.10	0.15	0.47	1	0.491	1.11
Prior Research Experience (1)	0.55	0.47	1.37	1	0.242	1.73
Intercept	−0.65	0.77	0.72	1	0.398	0.52
Overall Model Test	*Χ*^2^(11) = 12.30, *p* = 0.342					

**Table 15:** Master’s enrollments by program and cohort year.

Cohort Year	Fellows (*n* = 64)	Scholars (*n* = 53)
2019	6	5
2020	6	3
2021	5	3
2022	7	5
2023	0	2
**Total**	**24/64 (37.5%)**	**18/53 (34.0%)**

*Note*: NSC data pulled 7 October 2025, supplemented by program data.

**Table 16:** Logistic regression results for master’s degree program enrollment and degree attainment (*N* = 117).

Predictor	Model 1 (Master’s Enrollment)						Model 2 (Master’s Degree Attainment)					
	*b*	S.E.	Wald	*df*	*p*	Exp(B)	*b*	S.E.	Wald	*df*	*p*	Exp(B)
BUILD Program (Fellow =1)	0.28	0.43	0.43	1	0.511	1.33	0.85	0.57	2.23	1	0.135	2.34
Transfer Student	−0.70	0.50	1.93	1	0.165	0.50	−0.17	0.64	0.07	1	0.796	0.85
Women	−0.23	0.47	0.24	1	0.626	0.80	0.06	0.60	0.01	1	0.915	1.07
First Generation	0.58	0.49	1.38	1	0.241	1.78	0.35	0.59	0.35	1	0.555	1.42
Pell Eligible	0.60	0.48	1.61	1	0.205	1.83	0.56	0.61	0.85	1	0.357	1.75
Discipline- Behavioral	0.89	0.47	3.63	1	0.057	2.42	1.58	0.64	6.16	1	0.013	4.87
Race/Ethnicity (Ref = Latine)			1.68	3	0.642				2.86	3	0.413	
Asian	0.43	0.53	0.64	1	0.424	1.53	1.08	0.66	2.71	1	0.100	2.96
White	0.59	0.62	0.90	1	0.342	1.79	0.58	0.78	0.56	1	0.454	1.79
Other Race/Ethnicity	−0.26	0.79	0.11	1	0.744	0.77	0.21	0.99	0.04	1	0.836	1.23
Program Year (Centered)	−0.18	0.15	1.56	1	0.212	0.83	−0.65	0.22	8.81	1	0.003	0.52
Prior Research Experience (1)	−0.02	0.44	0.00	1	0.964	0.98	0.35	0.59	0.36	1	0.551	1.42
Intercept	−1.63	0.77	4.53	1	0.033	0.20	−4.17	1.16	13.02	1	<0.001	0.02
Overall Model Test	*Χ*^2^(11) = 12.24, *p* = 0.346						*Χ*^2^(11) = 21.86, *p* = 0.025					
∆*Χ*^2^ (Model 2-Model 1)	∆*Χ*^2^(1) = 0.16, *p* = 0.689						∆*Χ*^2^(1) = 1.78, *p* = 0.182					
Psuedo-*R*^2^	0.14						0.27					

**Table 17:** GPA regressed on BUILD Program and controls (*N* = 117).

Predictors	*b*	S.E.	*B*	*t*	*p*
BUILD Program (Fellow = 1)	−0.01	0.05	−0.02	−0.20	0.844
Transfer Student	−0.08	0.06	−0.14	−1.48	0.143
Women	−0.15	0.05	−0.25	−2.81	0.006
First Generation	−0.03	0.06	−0.05	−0.57	0.572
Pell Eligible	−0.10	0.05	−0.18	−1.94	0.056
Discipline-Behavioral	0.26	0.05	0.45	5.03	<0.001
Asian	0.03	0.06	0.04	0.44	0.659
White	−0.04	0.07	−0.05	−0.55	0.580
Other Race/Ethnicity	−0.16	0.08	−0.16	−1.84	0.069
Program (Centered)	0.00	0.02	−0.02	−0.27	0.790
Prior Research Experience (1)	0.04	0.05	0.06	0.73	0.468
Intercept	3.72	0.09		43.63	<0.001
Overall Model Test	*F*(11,105) = 4.29, *p* < 0.001, *R*^2^ = 0.31				

## Data Availability

The data analyzed in this study is subject to the following licenses/restrictions: The dataset includes sensitive information including school records over multiple institutions, that could identify the subjects of this research study, especially for individuals from underrepresented minority groups, given the specificity of the information, including demographics and school record information, even when the data are scrubbed of names and ID numbers. Requests to access aggregate datasets should be directed to the corresponding author.

## Appendix A

**Table A1:** Items for psychosocial constructs.

Construct	Items
Perceived Research Skills (18 Items)	I have a skill in interpreting results
	I have a tolerance for obstacles faced in the research process
	I understand how knowledge is constructed
	I understand the research process in my field
	I have the ability to integrate theory and practice
	I understand how scientists work on real problems
	I understand that scientific assertions require supporting evidence
	I have the ability to analyze data and other information
	I understand science
	I have learned laboratory techniques
	I have the ability to read and understand primary literature
	I have the skill to give an effective oral presentation
	I have skills in science writing
	I have self-confidence
	I understand how scientists think
	I have the ability to work independently
	I am part of a Learning Community
	I have a clear understanding of the career opportunities in science
Science Identity (3 items)	I see myself as a scientist
	Science is important to me
	It is important to me that others see me as a scientist
Researcher Identity (3 items)	I see myself as a researcher
	Research is important to me
	It is important to me that others see me as a researcher
CSULB Belonging (3 items)	I “fit in” well at CSULB
	I have friends at CSULB
	I feel connected to the CSULB faculty
Program Belonging (4 items)	I see myself as part of the BUILD community.
	I feel connected to members of the BUILD community
	Being a member of the BUILD community is important to me
	I feel a sense of belonging within the BUILD community
Professional Belonging (4 items)	I feel confident that I will “fit in” in a Ph.D. program
	Sometimes I worry that I will not belong in a Ph.D. program (Reverse coded)
	I feel confident that I will “fit in” in the professional community of my discipline
	Sometimes I worry that I will not belong in the professional community of my discipline (Reverse coded)
Cultural Compatibility (10 items)	My cultural background is compatible with my goals of going to graduate school in my area of study (or discipline)
	My cultural background is compatible with my future career
	My cultural background has affected my success in my field in a positive way
	My cultural background will/would affect my success in a graduate program in a positive way
	My cultural background will/would affect my success in a biomedical/behavioral health-related career in a positive way
	Sometimes I feel I need to emphasize one aspect of my cultural background over another to fit into the academic community in my discipline (Reverse coded)
	Sometimes I feel I need to emphasize one aspect of my background over another to fit into different places or situations (Reverse coded)
	Sometimes I feel as though I need to hide parts of myself in order to succeed in my field (Reverse coded)
	Sometimes I feel as though I need to change the way I speak in order to succeed in my field (Reverse coded)
	Sometimes I feel as though I need to change the way I act in order to succeed in my field (Reverse coded)
Family Support (8 items)	I have talked to my family about the importance of science.
	I have had conversations with my family about career choices and educational plans.
	My family understands what I do in the BUILD Program
	My family supports what I do in the BUILD Program
	My family supports my plan to attend graduate school.
	My family responsibilities are different than those of my classmates (Reverse coded)
	My family fully supports my educational goals
	My family responsibilities and activities conflict with my educational responsibilities (Reverse coded)
Personal Resource Management Skills (5 items)	I am able to meet deadlines and hand work in on time
	I am able to use a planner, to-do list, or timetable to help me plan my work
	It is easy for me to prioritize what I need to accomplish
	I am aware of when I work or study best (e.g., morning, evening)
	I am able to balance all of my responsibilities (e.g., research, classes, school, home, family, friends)

**Table A2:** Pooled correlations for all psychosocial constructs at the end of the training program (*N* = 117).

Psychosocial Construct		1	2	3	4	5	6	7	8	9
1. Composite Research Skills	Pearson Correlation	1								
	Sig. (2-tailed)	-								
2. Composite Science ID	Pearson Correlation	0.55 ***	1.00							
	Sig. (2-tailed)	<0.001	-							
3. Composite Research ID	Pearson Correlation	0.51 ***	0.68 ***	1.00						
	Sig. (2-tailed)	<0.001	<0.001	-						
4. Composite CSULB Belonging	Pearson Correlation	0.29 *	0.29 *	0.40 ***	1.00					
	Sig. (2-tailed)	0.017	0.023	<0.001	-					
5. Composite Cultural Compatibility	Pearson Correlation	0.21, ns	0.16, ns	0.24, ns	0.39 **	1.00				
	Sig. (2-tailed)	0.099	0.241	0.051	0.002	-				
6. Composite Prof Belonging	Pearson Correlation	0.35 **	0.15, ns	0.22, ns	0.35 **	0.56 ***	1.00			
	Sig. (2-tailed)	0.006	0.290	0.089	0.004	<0.001	-			
7. Composite Family Support	Pearson Correlation	0.34 **	0.26, ns	0.19, ns	0.29 *	0.42 ***	0.36 **	1.00		
	Sig. (2-tailed)	0.003	0.052	0.114	0.024	<0.001	0.004	-		
8. Composite Time Management	Pearson Correlation	0.28 *	0.11, ns	0.08, ns	0.05, ns	0.05, ns	0.26 *	0.15, ns	1.00	
	Sig. (2-tailed)	0.036	0.456	0.581	0.711	0.728	0.050	0.275	-	
9. Composite BUILD Belonging	Pearson Correlation	0.25 *	0.33 *	0.43 ***	0.57 ***	0.30 *	0.29 *	0.43 ***	0.04, ns	1.00
	Sig. (2-tailed)	0.041	0.014	<0.001	<0.001	0.034	0.026	<0.001	0.807	-

*Note.* * *p* < 0.05, ** *p* < 0.01, *** *p* < 0.001; ns = nonsignificant.
